# Dentin-Derived-Barrier Membrane in Guided Bone Regeneration: A Case Report

**DOI:** 10.3390/ma14092166

**Published:** 2021-04-23

**Authors:** Jeong-Kui Ku, In-Woong Um, Mi-Kyoung Jun, Il-hyung Kim

**Affiliations:** 1 Department of Oral and Maxillofacial Surgery, Section of Dentistry, Armed Forces Capital Hospital, Seongnam, Saemaul-ro 117, Seongnam 13574, Korea; kujk123@gmail.com (J.-K.K.); haonflower@gmail.com (I.-h.K.); 2 R&D Institute, Korea Tooth Bank, 622 Eonju-ro, Gangnam-gu, Seoul 06101, Korea; 3 Sae·e Dental Clinic, 109-8, Songwon-ro, Jangan-gu, Suwon 16294, Korea; mijjomg@naver.com

**Keywords:** bone regeneration, collagen, dental implant, dentistry, demineralized dentin matrix, membrane

## Abstract

An autogenous, demineralized, dentin matrix is a well-known osteo-inductive bone substitute that is mostly composed of type I collagen and is widely used in implant dentistry. This single case report describes a successful outcome in guided bone regeneration and dental implantation with a novel human-derived collagen membrane. The authors fabricated a dentin-derived-barrier membrane from a block-type autogenous demineralized dentin matrix to overcome the mechanical instability of the collagen membrane. The dentin-derived-barrier acted as an osteo-inductive collagen membrane with mechanical and clot stabilities, and it replaced the osteo-genetic function of the periosteum. Further research involving large numbers of patients should be conducted to evaluate bone forming capacity in comparison with other collagen membranes.

## 1. Introduction

Since the osteo-genetic potential of the periosteum became known in the 19^th^ century, membranes have been developed to place the periosteum into bone defects [1,2]. Nonabsorbable membranes, introduced earlier than absorbable membranes, have a rigid mechanical property for good space maintenance and bone formation, but their disadvantages are susceptibility to infection when exposed and inevitable, additional surgery for removal [2,3]. There is excellent biocompatibility of absorbable membranes. On the other hand, they have been widely used for guided bone regeneration (GBR) despite having relatively poor mechanical properties [2]. Among the many types of absorbable membranes, collagen-based membranes have been developed based on the characteristics of collagen, which are its cell-attachment capability (RGD sequence), excellent tissue compatibility, absorbability, and weak antigenicity [4,5,6]. In vivo studies have shown that cells within the collagen membranes gradually express major bone-related growth factors [5]. However, no collagen membranes have been reported to have satisfactory mechanical stability and osteo-inductivity in clinical situations [2,3].

The components of bone and dentin are similar, and they include hydroxiapatite (70%), collagen (18%), noncollagenous proteins (2%), and body fluids (10%) in weight by volume [7]. Type I collagen is the main component in bone and dentin, which have similar chemical components [4,7]. A demineralized dentin matrix (DDM) from human teeth, which is widely used as a bone substitute in implant dentistry, consists of an avascular, acellular, dense, collagen matrix that contains non-collagenous growth factors, such as BMPs, [8] and has excellent osteo-inductivity and osteo-conductivity [9,10]. Block-type DDM has mechanical stability that could delay the remodeling process and endure bacterial invasion in conditions of exposure to the oral cavity [10]. Therefore, DDM is a potential candidate as an osteo-inductive collagen membrane to replace the periosteum as originally intended.

A block-type DDM (autogenous tooth bone graft block, ABTB, Korea Tooth Bank, Korea) has been shown to have excellent bone formation with biocompatibility and space maintenance in alveolar ridge augmentation and socket preservations [10,11,12,13]. A dentin-derived-barrier membrane (DDB) fabricated from the block-type DDM is characterized to have a resorbable, osteo-inductive, and collagenous nature, which has macro (0.2 mm) and micro (dentinal tubule) porosity. The purpose of this single case report was to present the possibility of DDB, which could be acting as a resorbable, bone-forming, collagen membrane.

## 2. Materials and Methods

This study was approved by the Institutional Review Board (No. AFCH-20-IRB-039) of our institution at 26 January 2021. A 62-year-old male with a noncontributory medical history presented with a non-savable left mandibular, first premolar, and first molar due to severe periodontal disease. The left mandibular, first molar, and the other molar were used as a DDB and DDM, respectively. Clinical photographs and a cone-beam computerized tomography (CBCT, Vatech, Seoul, Korea) scan were obtained. The patient was informed regarding the operative procedure and possible risks, and he signed an informed consent form.

### 2.1. Preparation of Dentin-Derived-Barrier Membrane (DDB)

The extracted first molar that was to be used for the DDB was immersed in 75% alcohol (Durvet, MO, USA). The tooth was then cleaned, and the remaining periodontal tissue was removed. After removing the soft tissues and calculus attached to the tooth, the root surface was carefully cleaned. Layers of enamel and cementum were removed using a rotating instrument (Diamond bur, Rodent AG, Seoul, Korea). The vital pulp tissue and a root canal filling were also removed. Additional holes with a diameter of 0.2 mm were made using a micro-fissure bur (Ø 0.2 mm, Bredent, Senden, Germany) from the dentin surface to the pulp chamber, and a root canal was formed to create macropores to promote vascular invasion and bone formation. Then, the tooth was processed for ABTB fabrication (European Patent No. 2462899) considering its intended use as described in previous reports [8,14]. Briefly, the ABTB was processed with demineralization, defatting, dehydration, and freeze-drying.

**Figure 1:**
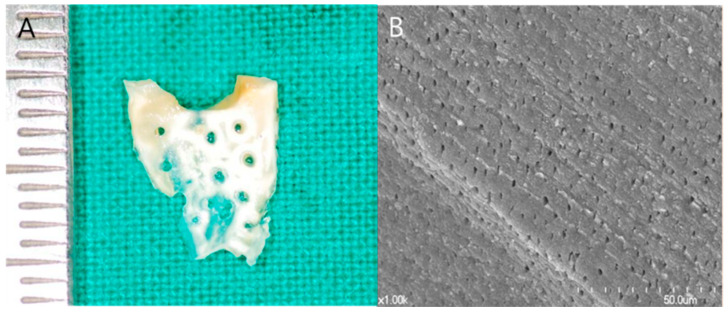
Dentin-derived-barrier membrane (DDB). (**A**) DDB was fabricated from a 300 to 800 µm thick slice of autogenous tooth bone graft block with 0.2 to 0.3 mm diameter holes. (**B**) Scanning electronic microscopy (S-4700, Hitachi, Tokyo, Japan) of the DDB surface with exposed dentinal tubules (×1000 magnification).

Subsequently, the processed ABTB was sliced in the occlusal–apical direction to form a 300 to 800 µm thick membrane (DDB, Figure 1). Sections were discarded if the pulp chamber was exposed during the slice procedure. Finally, DDB exhibited a tooth surface appearance with 0.2 to 0.3 mm diameter holes. The prepared DDB was dehydrated, freeze-dried, and stored at room temperature for a subsequent application.

### 2.2. Surgical Procedure (Application of a DDB)

**Figure 2:**
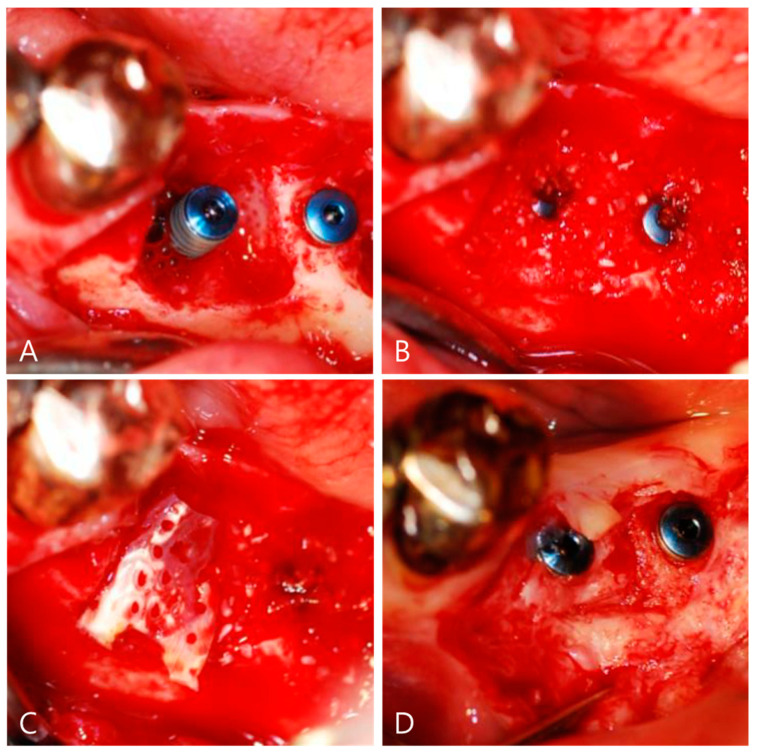
Surgical procedure for guided bone regeneration using a dentin-derived-barrier membrane (DDB). (**A**) Implants (3.8 mm in diameter, 13 mm in length, Dio, Busan, Korea) were placed with good primary stability. There was a buccal wall defect about 4 mm around the implant. (**B**) The defect around the implant was filled with autogenous bone graft materials (AutoBT powder, Korea Tooth Bank, Seoul, Korea). (**C**) The DDB covered the whole defect. The DDB showed a color change after soaking with the patient’s own blood due to the major collagenous nature. (**D**) The DDB had completely disappeared and was homogeneously incorporated at the graft site by re-entry at 14 weeks for a prosthetic procedure.

At four weeks after extraction, with the completion of the majority of soft-tissue healing [15], local anesthesia (4% Ubistesin^®^ with 1:200,000 adrenaline, 3M Espe AG, Seefeld, Germany) was applied. A careful incision and removal of all granulation tissue from the defect area were accomplished. Dental implants (diameter, 3.8 mm, length, 13 mm, Dio, Busan, Korea) were inserted in a slight supra-crestal position on the left mandibular premolar and first molar (Figure 2A). DDM was applied for socket preservation and ridge augmentation on the left mandibular premolar (Figure 2B). The DDB was positioned to carefully cover up the alveolar socket under controlled pressure to the height of the buccal and lingual alveolar bone (Figure 2C). Because the authors confirmed the stability of the DDB with autogenous blood, no additional fixing method was applied. The sutures were removed at 14 days after surgery. The patients did not report any discomfort, and wound healing was regular and uneventful. No clinical signs of significant infection or graft loss were present.

## 3. Results

**Figure 3:**
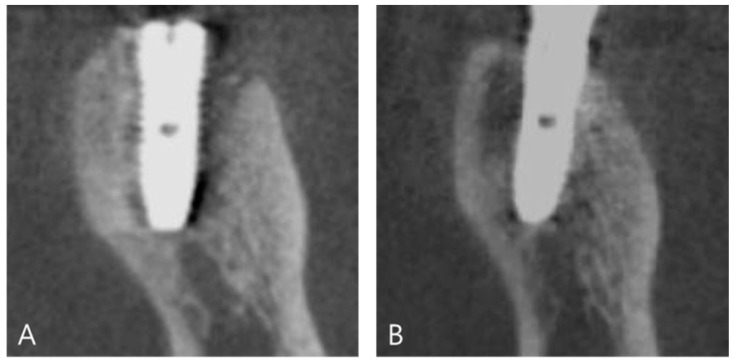
Cone-beam computed tomography after guided bone regeneration using a dentin-derived-barrier membrane (DDB). (**A**) Coronal plane of cone-beam computed tomography immediately after implant placement with guided bone regeneration using a DDB. There was a radiolucent defect that was filled with auto-DDM and covered with a DDB. (**B**) At 4 years and 8 months, the cortical bone around the implant neck was fully repaired and supported by well-developed corticocancellous bone.

At 14 weeks after surgery, the surgical site was reopened for a prosthetic procedure (Figure 2D). The DDB with DDM was transformed into newly homogeneous hard tissues surrounding the implant without a visible DDB. Prosthetic loading was performed at 17 weeks after the implantation. Consecutive follow-up examinations did not show any complications (Figure 3A). At 4 years and 8 months after prosthetic loading, the buccal defect, which was filled with DDM covering the DDB, and the crestal bone height were well maintained with sound corticocancellous bone on CBCT (Figure 3B).

## 4. Discussion

The aim of this case report is to present the possibility of DDB, which can act as a resorbable, bone-forming collagen membrane. Even though we presented only one case report of DDM application in GBR procedure, we achieved successful bone healing outcomes after a GBR procedure.

The ideal characteristics of a barrier membrane include biocompatibility, cell-occlusion properties, integration by the host tissues, clinical manageability, space-making ability, and adequate mechanical and physical properties [1]. Since repopulation of osteoprogenitor cells is known to be slower than that of fibrogenic cells, the membranes are used to block fibrogenic cells during the migration of osteoprogenitor cells in a GBR procedure [2]. On the other hand, many studies have been conducted to promote the regeneration of a grafted bone substitute under a membrane [2,3,4,5,6].

A collagen membrane is the most commonly used natural membrane in GBR because it can promote wound healing with cell–matrix interactions and bone formation that hosts various cell phenotypes, acts with an antibacterial effect, and is easily obtained from different species [1]. An in vivo study reported that cells recruited into the collagen membrane gradually expressed a signal for osteogenic factors, such as bone morphogenetic protein (BMP) [5]. Therefore, the membranes did not act only as a passive barrier, but they also had a functional role similar to the original periosteal function. To promote the regeneration process, human collagen-based membranes, such as skin and amnion membranes, have been tested [5,6]. However, there has been a lack of reports on the osteo-inductive functions of collagen membranes in clinical conditions. The strategies for administering growth factors with the membrane have provided promising experimental results. However, clinical evidence has yet to be provided for the growth factor strategy in conjunction with the membrane in GBR [3].

DDM is well known to have osteo-induction capacity with non-collagenous endogenous proteins binding to the dentin matrix [8,9,16]. The antigenicity of human dentin has been originally low because dentin is an acellular and avascular collagen matrix [14]. In addition, the demineralization process contributes to the enlargement of the dentinal tubules, which could affect the release of the dentin-matrix-derived (endogenous) growth factors, resulting in the activation of osteo-inductivity by phenotypic transformation of fibroblasts into osteoblasts [8,11,17,18,19].

Among many types of DDMs, the autogenous tooth bone graft block (ABTB, block-type DDM) is a biomimetic of cortical bone that showed promising clinical outcomes in socket preservation and ridge augmentations [13]. Although DDM has 3–6 µm micropores in an enlarged dentinal tubule, additional 100–200 µm macropores are created to promote osteo-genecity in the ABTB manufacturing process [10,13]. Consequently, ABTBs have especially shown the capacity to achieve secondary healing in an exposed condition, such as wound dehiscence [10,13].

DDB, being sliced with 300–800 µm thickness from ABTB, showed a natural Type 1 collagen membrane, which is biomimetic of cortical bone due to the remaining mineral component after demineralization [10]. The DDB has innate micropores (dentinal tubules) ranging from 3 to 5 µm and macropores ranging from 0.2 to 0.3 mm. Regarding the pore size of membranes that have been reported as essential for bone and soft-tissue regeneration by the diffusion of cells, growth proteins, and blood circulation with nutrients, a larger pore size generally shows better cell and nutrient invasion and tissue-occlusivity, but soft-tissue infiltration may also interrupt bone regeneration [1]. The optimal pore size and porosity have not yet been defined, and the relation between the DDM as graft materials and DDB as a barrier membrane should be considered. Although primary closure was achieved in this case, the DDB might have protected the grafted bone substitutes inside the DDB from infection like other collagen membranes. Furthermore, with the DDB, the bone formation of the grafted bone can be achieved as fast as the function of the periosteum due to the rapid remodeling capacity of the DDM with endogenous BMP from dentin [8].

In addition, a recent histological review of a demineralized dentin matrix as a carrier of rhBMP-2 implied that rhBMP-2 incorporated into DDM (rhBMP-2/DDM) might simultaneously initiate osteoclastic resorption of DDM and osteo-inductive bone healing [11]. The postulated release profile of rhBMP-2/DDM was suggested to be a sequential delivery of exogenous rhBMP-2 and the endogenous BMP in a physiological environment [8]. Therefore, DDB incorporated with rhBMP-2 might also have great potential by enhancing osteo-inductivity with the sequential delivery of rhBMP-2, and it might sustain the rhBMP-2 concentration for a prolonged period.

Some clinical studies have reported successful results on the allogeneic application of DDM [14,20,21,22,23,24,25,26,27,28,29,30,31,32]. Still, many clinical applications do not allow any interim considerations, including pooling, standardized fabrication, and a demineralization protocol, antigenicity, and optimal viral clearance methods as well as differentiation from a demineralized bone matrix. To facilitate the availability of DDB as a novel biomaterial that overcomes the limitation of autogenous tissue, further studies should be conducted on the use of allogeneic DDB to reveal its safety and efficacy.

Due to the limitation of this single case report to present the possibility of a clinical application of DDB, the concerns could not be performed for treatment alternatives, which is a comparison with other evidence-based results, such as the mechanical impact of biomaterials/TCPs on cell growth, biological behavior in other conditions, and the role of local stem cells in the local environment. Taken altogether, a general interpretation of the results in the context of other evidence and variables should be provided in future research [33,34,35,36,37].

## 5. Conclusions

A successful GBR could be achieved by employing a DDB that was fabricated from ABTB (block-type DDM) with a proven osteo-inductive property. Therefore, a DDB might be considered a new, natural collagen membrane in implant dentistry. Further studies, including randomized controlled trials involving large numbers of patients, should be conducted to evaluate bone forming capacity in comparison with other collagen membranes.

## Data Availability

Data are contained within the article.
